# Within-Tree Distribution and Survival of the *Eucalyptus* Longhorned Borer *Phoracantha semipunctata* (Coleoptera: Cerambycidae) in a Mediterranean-Type Ecosystem

**DOI:** 10.3390/insects11040225

**Published:** 2020-04-04

**Authors:** Stephen Seaton, George Matusick, Giles Hardy

**Affiliations:** Environmental and Conservation Sciences, Murdoch University, Melville 6150, Australia; g.matusick@murdoch.edu.au

**Keywords:** survival, *Phoracantha semipunctata*, within-tree, larval incidence, galleries, emergence

## Abstract

The attack patterns, infestation success and larval development of woodborers within living trees are complex and are largely shaped by host tree characteristics. Following a severe drought in a native eucalypt forest where outbreak densities of a native Australian beetle, the eucalyptus longhorned borer (*Phoracantha semipunctata)*, occurred, a tree dissection study was conducted in Australia. This involved felling 40 trees each of jarrah (*Eucalyptus marginata*) and marri (*Corymbia calophylla*) that were cut into 1-m sections and neonate larval galleries, larvae in pupal cells and adult borer emergence were measured and added to give total numbers per tree to determine the within-tree distribution and survival of *P. semipunctata*. There was a significant impact on larval survival in both species, in contrast, pupal survival remained high. Within-tree distribution of *P. semipunctata* was directional with borer emergence and incidence of larval galleries both negatively associated with tree section height above the ground and positively associated with section diameter and bark thickness, reaching a maximum towards the base of trees. High incidence and survival in lower thicker tree sections indicate a more conducive environment for larval development, in contrast to poor larval survival in smaller thinner sections at the top of trees. The dependence of larval survival on tree characteristics controlling the within-tree distribution of borer emergence is emphasized, and needs to be considered when estimating the spread of borer populations during outbreaks.

## 1. Introduction

The development of woodborer larvae is more restricted, compared to other phytophagous insects of defoliators, as they are concealed within their hosts and sapwood utilisation is determined by food resources emanating from the location of egg laying by their female parent [1,2]. This is unlike the larvae of defoliators that can move as required to more favourable and nutritious parts of the tree. The natural taper of a tree does, however, produce different developmental conditions [3,4] and can influence the ability of borer larvae to reach maturity. For instance, diameter, bark and phloem thickness are autocorrelated with height above the ground, and these contribute to the success of wood-boring insects, with a tendency for increased densities of within-tree populations towards the base of trees [5,6,7]. The great capricorn beetle *Cerambyx cerdo* Linnaeus (Coleoptera: Cerambycidae) in oak (*Quercus* spp.) prefer large-diameter tree sections occurring lower along the tree trunk that have been exposed to the sun [6], and higher densities of the Australian eucalyptus longhorned borer *Phoracantha semipunctata* Fabricius (Coleoptera: Cerambycidae) emerge when infesting larger diameter *Eucalyptus* logs in non-native habitat [8]. Bark thickness can affect the rate of larval growth [9] and promote regions of high density [5] by creating a more suitable sapwood environment for larval development. Differences in phloem nitrogen and thickness and available carbohydrates control the rate of larval growth and survival in phloem and xylem feeding borers [10,11,12]. For example, phloem density and low moisture content of *Corymbia citriodora* Hook (lemon scented gum) encouraged high borer densities of *P. solida* Blackburn [7]. However, although phloem thickness generally increases with diameter this does not influence woodborer attack density [13].

*Phoracantha semipunctata* is native to forests within Australia and parts of New Guinea [14,15,16]. In Australia, the species appears inconspicuous only contributing to a small proportion of insects caught in traps within eucalypt forests [17]. In addition, infestations causing widespread damage in Australia are rare, as attacks by this borer are restricted to trees severely affected by drought [18,19], and seldom reported as causing significant damage to trees. Although in Southwestern Australia it had been observed to kill young jarrah trees in revegetated bauxite mine pits [20], in Australia it is not considered a serious pest of *Eucalyptus* tree species. In comparison to countries in the Northern Hemisphere where it is not native, it is considered a major pest species and has become widespread in their eucalypt plantations [18,21]. In particular, it has caused significant economic damage in apparently healthy trees in Palestine, the United States [1,18] and Morocco [22]. Additionally, in countries in the Southern Hemisphere, outside of Australia it can be a pest [23], although it is not always considered a major pest.

Tree taper may alter the developmental conditions for borers and, in turn, affect larval survival. Borers can exhibit a host species preference, and in a study of *P. semipunctata* conducted overseas, logs of *Eucalyptus trabutii* Vilmorin with higher neonate densities produced a lower survival compared to *E. cladocalyx* F.von Müller, *E. grandis* Maiden and *E. tereticornis* Smith [24]. The nutritional quality of phloem/sapwood of eucalypts declines after hosts become stressed [25]. Intraspecific competition among high numbers of larvae was suggested to force the maturing larvae that were delayed in their development to feed on sapwood of lower quality [1,26,27]. In this environment, cannibalism is likely to occur between larval conspecifics, with increased mortality of smaller larvae [8,25,27,28]. Additionally, larvae that complete the majority of their development before cooler winters are suspected to be feeding on phloem/sapwood with higher nutritional quality, with larger adults emerging from these hosts [29]. These studies were conducted in young plantation trees or cut logs; however, little is known of *P. semipunctata* behaviour and development among hosts of whole eucalypt trees within native Australian ecosystems. Consequently, the survival of borers and total borer numbers emerging in a forest depends on how tree size and shape alter the conditions within the trees. This information is critical to understanding their extent of attack and for implementing control programs for this species in eucalypt forests and plantations.

Adults of *P. semipunctata* are 15–23 mm long and active throughout spring and summer, emerging from around dusk onwards, they feed on pollen and nectar of eucalypt flowers [21,26,30]. Adults are attracted to volatiles released by stressed and dying trees, they then mate and oviposit through cracks and crevices in loose bark [1,26,31,32]. Larvae initially begin consuming the outer layers of bark and phloem before they spread away from an oviposition site and consume the phloem and outer layers of sapwood [21,23,33]. As larval feeding continues, extensive galleries are produced and almost the entire phloem/sapwood of trees and logs can become riddled with galleries [8,27]. In some cases, the complete cambium zone can be consumed during larval development, eventually killing the tree [18,20,26,34], although mature larvae are known to avoid feeding near oviposition sites where phloem/sapwood may be dry [8]. Larvae then enter deeper into the tree by boring through the sapwood and occasionally enter the heartwood, where they form a pupal chamber. Adult beetles then emerge by eating through the frass-filled entry tunnel to the chamber and create an oval exit hole of around 8–10 mm in diameter through the bark surface [1,30]. At the time of pupation, the sapwood has dried significantly, and with reduced nutritional quality this can limit the ability of late-stage larvae to complete development and pupate [2,32]. In overseas plantations, its life cycle takes 8–18 months [1,18,35], but can be as little as two months in logs with high larval densities [8].

Large areas of stressed and dying trees associated with a drought event, leading to widespread attack by *P. semipunctata* in Southwestern Australia jarrah forest, showed that tree health was critical for determining the intensity of borer emergence [36]. To estimate the borer’s population size and better understand its behaviour, these drought-stressed trees in the Northern Jarrah Forest (NJF) provided an opportunity to determine how tree characteristics shaped the within-tree distribution and affected the survival of *P. semipunctata* in Australia. The study area dominated by jarrah (*E. marginata*) is a tree with exceptionally hard wood [37] belonging to the stringybarks with rough elongated fibrous bark [38]. In contrast marri (*C. calophylla* L.A.S. Johnson) has softer wood and belongs to the bloodwoods [39,40] with rough tessellated bark [38]. Both species are the main overstorey trees native to the NJF.

## 2. Materials and Methods

### 2.1. Study Area and Tree Selection

The study was conducted in the NJF in the Darling Range, Southwestern Australia. This region comprises one of the five biodiversity hotspots in Mediterranean climate regions [41,42] and experiences cool, wet winters (rainfall from 800–1100 mm) followed by dry summers lasting up to six months, where total rainfall can be <50 mm [43]. In the NJF, jarrah and marri comprise the main overstorey species that can reach 30 m high with an understorey of *Banksia grandis* Willd, *B. attenuata* R.Br., *B. littoralis* R.Br., *Allocasuarina fraseriana* Miq. and grass trees (*Xanthorrhoea preissii* Endl.) with ground cover composed of many species of shrubs and herbaceous perennials [44].

A severe drought and heat-wave event in 2010/2011 resulted in patches of forest suffering severe canopy die-off [45] and subsequent attack by *P. semipunctata* [36]. In March–April 2012 following the die-off event, four of these die-off patches were used, two were located in the West-central jarrah forest (32°13′36.96″ S, 116°8′1.24″ E), and two sites 40-km (32°34′32.40″ S, 116°0′53.11″ E) to the south. Within each site, 10 jarrah and 10 marri trees with no live canopy present were selected and were at sufficient distance apart to account for variations throughout the whole site.

### 2.2. Tree Characteristics and Borer Life Stages

Sample trees were felled at ground level, total tree height measured and then divided into 1-m long sections (average surface area of 0.458 m^2^) and numbered accordingly to study the within-tree distribution of *P. semipunctata*. Diameters were measured at the midpoint of each section (giving 861 stem sections) to at least ≥5 cm in diameter. An estimate of bark surface area for a cylinder 0.5 m above and below the mid-point of each tree section was then derived from the diameter of each section. Summing these estimates gave an approximation of the total bark surface area of each tree. A 5-cm limit was set, as in some sections, not all the wood smaller than this size could be recovered after felling. Bark thickness was measured at the distal end of each section at three points around the circumference.

Within each tree, measurements were made for the developmental life stages of *P. semipunctata*, namely; oviposition sites, neonate larval galleries, prepupal larvae in pupal cells and adult borer emergence (measured as emergence holes). For every 1-m section, the loose outer bark was removed to ensure all emergence holes made by emerged borers could be clearly located and were then counted. These holes were oval in shape and approximately 8 mm in diameter; with adult borers present in some of these holes were identified as *P. semipunctata* using taxonomic keys [14,18,46]. No other species of Cerambycidae were found. Next, the inner bark was removed from every odd numbered 1-m section to count oviposition sites and neonate larval galleries present in the exposed sapwood surface. Only every odd section was measured for these parameters as tests on several trees for each species where all sections of bark were removed showed an error that averaged 2.0%. In other studies, on the borer *E. rufulus*, this method of subsampling half of the tree bark sections were found to give the lowest error for estimates of galleries and larvae of the whole tree [47]. Oviposition sites were identified by narrow feeding galleries made by neonate larvae radiating out from a central point. In some cases, oviposition sites and neonate larval galleries could not be distinguished as they were obscured by late-stage larval galleries and these samples were excluded from analyses.

To assess the intensity of larval activity the incidence of larval galleries was measured. This was achieved by wrapping a 100 cm^2^ transparent grid around the exposed sapwood surface at three random positions for each odd numbered 1-m section, and the number of 1 cm^2^ squares that were intersected by at least one larval gallery was counted. Counts were then averaged and expressed as a percentage for each section. Sections were then longitudinally split a number (1–5) of times, depending on their diameter, to reveal pupal chambers in the inner sapwood and heartwood and all prepupal larvae within pupal chambers were counted.

Emergence holes for all 1-m sections were added to give total numbers of borer emergence per tree. Oviposition sites and neonate larval galleries for each odd section were doubled and then added to give total numbers per tree. Density (m^−2^) of borer emergence for each 1-m section was calculated by dividing the number of emergence holes in a section by the surface area over the bark of that section. Density (m^−2^) of oviposition sites and neonate larval galleries for each odd section were calculated by dividing numbers in each odd section by the surface area over the bark of that section. Densities (m^−2^) per tree of oviposition sites, neonates and borer emergence were calculated by averaging the density values of all sections for the whole tree. Gallery incidence per tree was calculated by averaging incidence (%) of each section in a tree. Larval survival per tree was calculated by dividing the total number of larvae that had at least reached the pupal stage by the total number of neonate larval galleries; pupal survival was calculated by dividing the total number of emergence holes by the total number of larvae that had at least reached the pupal stage, and total survival of adult borers by dividing the total number of emergence holes by total number of neonate larval galleries.

### 2.3. Statistical Analyses

Comparisons of average density (m^−2^) of oviposition sites, neonate larval galleries, late-stage larvae and borer emergence, and percent (%) incidence of larval galleries for each tree were tested for differences between tree species with Analysis of Variance (ANOVA) using Genstat v18 [48]. Percent (%) total, neonate, and pupal survival for each tree were tested for differences between tree species with ANOVA. For each ANOVA, to determine if sample data were normally distributed, checks were visually made using standardized residual plots, normal probability plots and the W or Shapiro–Wilk test. Additionally, Levene tests for stability of variance were conducted. Density data were square root transformed and percentage data arcsine transformed where necessary to achieve normality with means ± SEM shown where appropriate.

Analyses of the association of (a) numbers of borers emerged with height (m) and diameter (cm), (b) density of borer emergence (m^−2^) with height per section, and (c) incidence of larval galleries (%) with height (m) and diameter (cm) were tested using separate generalised linear mixed models (GLMM). For models (a) and (b), data for the borer variate were Poisson transformed with logarithm link function while for model (c) data for incidence (%) variate were arcsine transformed. For all models, the tree nested within site was included as the random effect. For the first model, the borer variate was the number of borers that emerged, and the fixed effects were species, height, and section diameter and their interactions. In the second model, the variate was borer emergence (m^−2^), and the fixed effects were species and height and their interactions. For the third model, the borer variate was incidence of larval galleries (%), and the fixed effects were species, height, and section diameter and their interactions. Bark thickness of sections was grouped into classes that increased by 2 mm from the smallest to largest values and averaged for each class for each tree species, and only those 2 mm classes containing three or more values were included in the analysis.

Relationships of diameter (cm) with height per section (m); bark thickness (mm) with diameter; borer emergence (m^−2^), incidence of larval galleries (%) and larval survival (%) with height per section; borer emergence (m^−2^) and incidence of larval galleries (%) with bark thickness (mm) and also incidence of larval galleries (%) with neonate larval galleries per tree (m^−2^), for each tree species were compared using least squares regression in Genstat v18, testing for differences in slope via likelihood ratio statistics for common slopes between tree species, and elevation by Wald statistics assuming common slope for each tree species.

## 3. Results

### 3.1. Tree Characteristics and Borer Emergence

**Figure 1:**
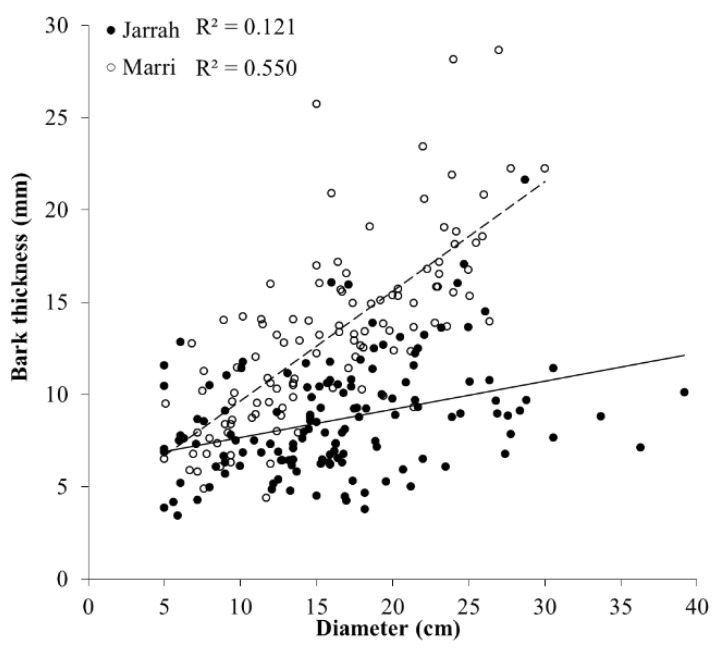
Relationships between bark thickness (mm) and diameter (cm) for 1 m sections up to 13 m in height for jarrah (*Eucalyptus marginata* ●) (*n* = 142) and marri (*Corymbia calophylla* ○) (*n* = 114) trees infested by the Australian eucalyptus longhorned borer *Phoracantha semipunctata* in the Northern Jarrah Forest (NJF), Southwestern Australia.

Diameter (cm) per section decreased with increasing height (m) in jarrah and marri (*F*_22,830_ = 24.37, *p* < 0.001), where marri decreased significantly more (1.41X) in slope with height compared to jarrah (*χ*^2^ < 0.001) and marri had higher elevation for a given diameter (*χ*^2^ < 0.001) (Table 1). At a 7-m height, diameter of marri was significantly smaller than jarrah (*F*_22,830_ = 13.49, *p* < 0.001). Bark thickness (mm) per section also decreased (*p* < 0.001) with height, with both species having similar slopes (*χ*^2^ = 0.106), and marri had higher elevation for a given section height (*χ*^2^ < 0.001) (Table 1). Bark thickness per tree was greater (1.4X) in marri at 12.79 ± 0.57 mm than jarrah at 9.13 ± 0.51 mm (*F*_1,79_ = 64.88, *p* < 0.001). For each tree species, bark thickness significantly increased with diameter (*F*_2,247_ = 85.96, *p* < 0.001), where marri increased significantly more (4.2X) in slope with diameter compared to jarrah (*χ*^2^ < 0.001), and marri had higher elevation for a given bark thickness (*χ*^2^ < 0.001) (Table 1, Figure 1).

Tree diameters at breast height (DBH) averaged 20 ± 1.0 cm (range 10–40 cm), and height (measured up to the position in the middle of the upper crown) averaged 11.3 ± 0.3 m tall (range 7–16 m). Tree species were similar in height (*F*_1,79_ = 0.01, *p* = 0.929), DBH (*F*_1,79_ = 0.32, *p* = 0.575), surface area (5.174 ± 0.281 m^2^, *F*_1,79_ = 0.32, *p* = 0.575) and basal area (0.035 ± 0.004 m^2^, *F*_1,79_ = 0.98, *p* = 0.326), from analysis by ANOVA.

**Figure 2:**
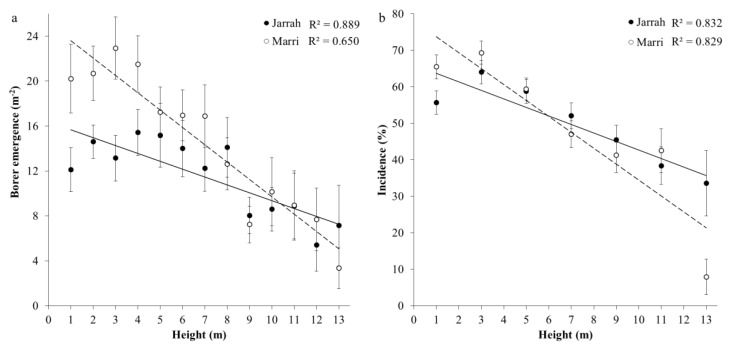

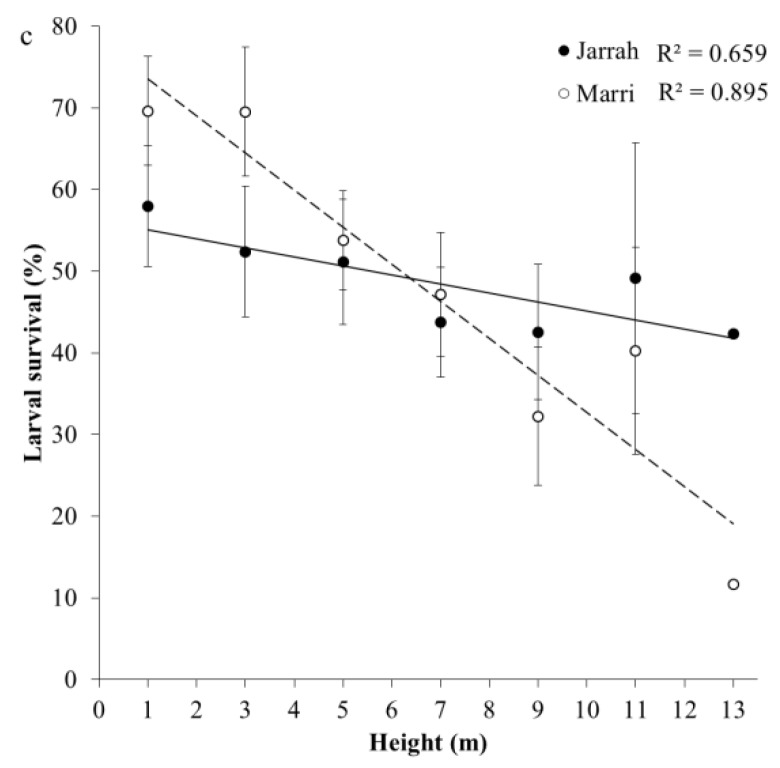
Relationship between mean (±SEM) (**a**) density of borer emergence (m^−2^), (**b**) incidence of larval galleries (%) and (**c**) larval survival (%) (neonates to larvae that had at least reached the pupal stage) of the Australian eucalyptus longhorned borer *Phoracantha semipunctata* with height (m) of 1 m sections up to 13 m in height for jarrah (*Eucalyptus marginata* ●) (*n* = 38) and marri (*Corymbia calophylla* ○) (*n* = 39) trees in the Northern Jarrah Forest (NJF), Southwestern Australia.

Borers emerged from all 80 trees sampled of jarrah and marri, and the total number of borers that emerged ranged from 5 to 429 per tree. Borer emergence was concentrated in the lower third of the trees, where on average 93% of sections contained an emergence hole, and half of the total borers that emerged from jarrah occurred up to a 4-m height, while for marri, this occurred between 2–3 m height. Adjusting for bark area, average density (m^−2^) of borer emergence per tree was significantly higher (33%) in marri at 16.57 ± 1.55 m^−2^ than for jarrah at 12.11 ± 1.37 m^−2^ (ANOVA, *F*_1,75_ = 6.08, *p* = 0.016). Borer emergence (m^−2^) decreased significantly with section height of trees (*F*_7,51_ = 13.83, *p* < 0.001) (Figure 2a, Table 2) and as slopes were not significantly different between sites they were combined for analyses (Likelihood ratio = 4.314, *χ*^2^ = 0.229). Borer emergence (m^−2^) decreased from 15.64 to 7.98 m^−2^ for jarrah and 23.29 to 7.22 m^−2^ for marri over a height of 13 m (Figure 2a), where there were similar slopes between species (*χ*^2^ = 0.164) and marri had significantly higher elevation than jarrah (*χ*^2^ = 0.001) (Figure 2a). For all sections in the lower half (7 m) of the trees, borer emergence in marri was significantly higher (40%) than jarrah (*p* < 0.001) (Figure 2a), where tree species were of similar diameters (12 to 15 cm), and bark of marri was 52% thicker (*p* < 0.05) than jarrah.

Incidence of larval galleries (%) (measured by the number of squares containing a gallery in a 10 × 10 cm^2^ grid) was negatively correlated with height (*F*_5,479_ = 24.95, *p* < 0.001) (Figure 2b, Table 2). Gallery incidence (%) was significantly higher for all sections in the lower 5-m of the trees (average 61.43 ± 3.13%) compared to sections above 7 m (average 40.51 ± 2.77%), although tree species had similar slopes (*χ*^2^ = 0.192) and elevation (*χ*^2^ = 0.808) (Figure 2b). For all sections at each height with incidence > 75%, residuals of gallery incidence remained constant at 8.52% between each 1 m height (ANOVA, *F*_6,144_ = 0.29, *p* = 0.939). Average gallery incidence per tree was also similar between tree species (ANOVA, *F*_1,75_ = 0.05, *p* = 0.815) with jarrah 47.5 ± 3.62%, and marri 49.0 ± 3.58%.

Survival of neonate larvae in marri was negatively correlated with height (*F*_5,479_ = 35.75, *p* < 0.001) (Figure 2c, Table 2).

Emergence and incidence data were analysed using a GLMM procedure for non-normal data. For the first model, the number of borers that emerged was significantly dependent on species, and negatively correlated with the height of a tree section above ground (m) and positively correlated with section diameter (cm), with significant interactions except for species and diameter (Table 3). A second model, adjusting for bark area (m^2^) of each 1-m section, showed that the density of borer emergence (m^−2^) was significantly dependent on tree species, and negatively correlated with the height of a tree section, with a significant interaction between height and diameter (Table 3). For the third model, incidence was not dependent on species and negatively correlated with height and positively correlated with section diameter (cm), with a significant negative interaction with height and diameter (Table 3).

**Figure 3:**
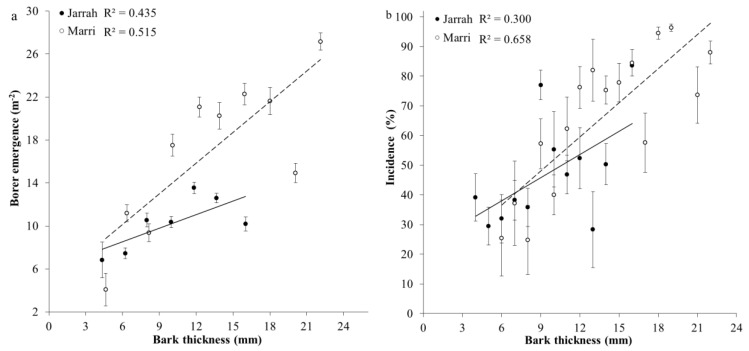
Relationship between mean (±SEM) (**a**) density of borer emergence (m^−2^) and (**b**) incidence of larval galleries (%) of the Australian eucalyptus longhorned borer *Phoracantha semipunctata* and bark thickness for jarrah (*Eucalyptus marginata* ●) (*n* = 38) and marri (*Corymbia calophylla* ○) (*n* = 39) trees in the Northern Jarrah Forest (NJF), Southwestern Australia. Data are presented within 2-mm bark thickness intervals.

Borer emergence significantly increased with bark thickness (*F*_5, 247_ = 11.97, *p* < 0.001) with similar slopes for regressions between species (*χ*^2^ = 0.583) and marri had significantly higher elevation for a given density of borer emergence compared to jarrah (*χ*^2^ = 0.018), (Figure 3a, Table 2). At a bark thickness of 16 mm, borer emergence was significantly (*p* < 0.05) higher (1.5X) in marri compared to jarrah (Figure 3a). Incidence significantly increased with bark thickness (*F*_5, 247_ = 13.36, *p* < 0.001) with jarrah and marri having similar slopes (*χ*^2^ = 0.719) and elevations (*χ*^2^ = 0.090) (Figure 3b, Table 2).

### 3.2. Incidence of Larval Galleries in Sapwood and Survival

**Figure 4:**
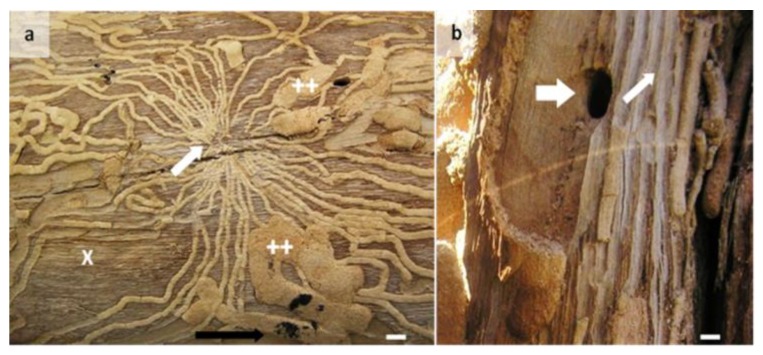
Photos of an example of galleries in surface sapwood of marri (*Corymbia calophylla*) infested by the Australian eucalyptus longhorned borer *Phoracantha semipunctata* showing: (**a**) High numbers of neonate larval galleries radiating out from a central oviposition site (white arrow) overlaid by late-stage larval galleries (++) leaving areas uneaten (x) with the tree axis left to right (black arrow), and; (**b**) An emergence hole made by an adult *P. semipunctata* (big arrow) and the thin partition of sapwood (small arrow) between galleries of individual larvae. White scale bars = 5 mm.

Density (m^−2^) of oviposition sites per tree were not significantly different between species (*F*_1,75_ = 0.79, *p* = 0.377) with jarrah 2.80 ± 0.19 m^−2^, and marri 2.58 ± 0.17 m^−2^. Additionally, density (m^−2^) of neonate larval galleries per tree were not significantly different between species (*F*_1,75_ = 1.85, *p* = 0.178) with jarrah 22.37 ± 1.86 m^−2^ and marri 25.30 ± 2.04 m^−2^, nor was density of late-stage larvae (*F*_1,75_ = 0.64, *p* = 0.426). Patterns of neonate feeding of phloem/sapwood by *P. semipunctata* radiated out from a confined oviposition site, then tended to orientate along the length of the tree, missing areas at right angles to the length of the tree (Figure 4a). As larvae developed, continued consumption of phloem/sapwood generally produced galleries that extended for at least 60 cm in parallel lines until larvae had reached maturity. Galleries occasionally crossed over galleries previously made by other developing larvae in the same area of surface sapwood, and in some cases, resulted in a loss of the thin partition left between galleries made by individual larvae, while other areas of surface sapwood surrounding oviposition sites were left uneaten (Figure 4b). There were significant (*p* < 0.001) positive relationships for gallery incidence with density of neonate larvae, reaching up to 85.1 ± 3.06% and 80.3 ± 1.25% incidence for jarrah and marri, respectively. Regressions for jarrah *y* = 0.855*x* + 28.38, and marri *y* = 1.323*x* + 14.37 having similar slopes (*χ*^2^ = 0.262) and elevations (*χ*^2^ = 0.773).

**Figure 5:**
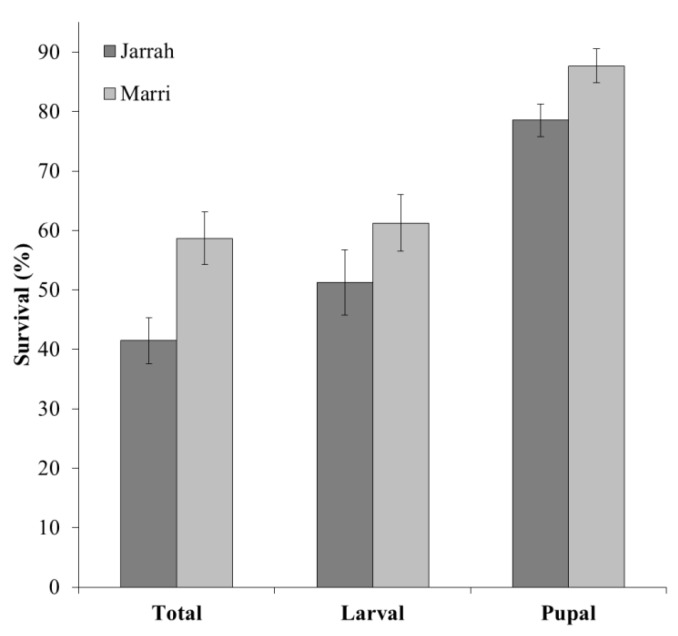
Percent survival of the Australian eucalyptus longhorned borer *Phoracantha semipunctata* for jarrah and marri trees, showing mean (±SEM) total survival (neonate larvae to emerged borers), larval survival (from neonates to pupation) and pupal survival (larvae that reached pupation to emerged borers) (*n* = 35, 37, 34, 36, 34 and 36 trees, left to right).

Total survival (neonate larvae to emerged borers) per tree averaged 50.3 ± 4.1% across species and was significantly higher (1.4X) in marri than jarrah (*F*_1,75_ = 9.55, *p* = 0.003) (Figure 5). This loss was mainly due to neonates failing to survive to maturity (neonates to late-stage larvae) and averaged only 56.4 ± 5.1% across species, with species not significantly different (*F*_1,75_ = 2.20, *p* = 0.142). Pupal survival (late-stage larvae to emerged adults) averaged 83.3 ± 2.8% across species and was significantly higher than neonate survival (*p* < 0.05) (1.5X). In marri, pupal survival was significantly higher than jarrah (*F*_1,75_ = 8.37, *p* = 0.005) (1.1X) (Figure 5).

## 4. Discussion

Approximately half the neonate larvae in both tree species survived to maturity, indicating the stability of the larval environment was affecting the development of *P. semipunctata*. This was evident with high gallery incidence and the formation of galleries that overlapped each other destroying the thin partition of sapwood that separated individual larvae during development. This suggests aggressive interference competition (conspecific facultative predation) was occurring [49,50,51]. Younger larvae may have become more susceptible to cannibalism by more mature larvae [8,25,27,28], and larvae that had been delayed in their development were potentially eating the frass made by older larvae [2]. However, feeding was concentrated in certain areas of sapwood, while in other areas, sapwood remained largely uneaten. Larvae may have missed these areas of sapwood due to variation in phloem density within the tree [7,11]. Areas of uneaten sapwood may also be related to the linear nature of gallery formation that forced larvae to feed in more confined areas of sapwood increasing the likelihood of competition among conspecifics. Phloem/sapwood tissues also decrease in quality after tree death [1,26,27], and later developing larvae could not compete for limited sapwood resources that at the time were likely to have also been low in nutrition. Cannibalism from larvae trying to consume diminishing food resources (exploitative competition) during development was also found for *P. semipunctata* on eucalypts in Africa [25] and is an important ecological trait held by other Cerambycidae [52,53,54]. Other factors that may have contributed to the poor survival, although were not investigated, include parasitoids and predators [2], poor nitrogen content levels of eucalypts [11,55], thickness of phloem [7], and carbohydrate and cellulose levels [56].

The high pupal survival (above 80%) of *P. semipunctata* in its native Australian habitat is in contrast to the low proportion of mature larvae *P. semipunctata* in a non-native habitat that did not finish constructing pupation chambers [8,27]. Other Cerambycidae have failed to complete pupation [57] and this may be due to unsuitable microclimate conditions and microbial infection [8]. Additionally, within the native range of *P. semipunctata* in the NJF the lower pupal survival in jarrah compared to marri may be related to jarrah wood being 20% harder than marri [40,58], which may have exhausted larvae as they may not have had enough energy to excavate pupal tunnels, form pupal chambers in dry nutrient poor heartwood [8] of jarrah, survive pupation, and emerge.

The dependence of increased gallery incidence, improved survival and greatest borer emergence of *P. semipunctata* in basal tree sections, having larger diameters and thicker bark, indicates the sapwood environment in these parts of trees are more conducive for borer development. The influence of bark thickness was more pronounced where the thicker bark in marri compared to jarrah may have contributed to higher borer emergence, with some indication of improved larval survival, despite lower sections of both tree species having similar diameters. Additionally, differences in bark structure between tree species may have modified the within-tree environment, with grooves and fissures in jarrah formed by having stringybark compared to the tessellated bark in marri [38], creating more gaps and allowing moderation of airflow, temperature and humidity for developing larvae. Lower regions of trees are important for producing the highest densities of several Cerambycidae, with a sawyer beetle (*Monochamus sutor* Linnaeus) in larch, red oak borer (*Enaphalodes rufulus* Haldeman) and great capricorn beetle (*C. cerdo*) in oaks and a buprestid the emerald ash borer (*A. planipennis*) in ash [5,6,59,60]. Thicker bark may have provided a more stable and cooler environment within the sapwood, with reduced desiccation and protection from predators, and was suggested to be associated with increased densities of larvae and adult emergence of other Cerambycidae [5].

Reduced larval survival in upper sections of jarrah and marri trees indicates unfavourable conditions were limiting the ability of borer larvae to complete their development. These upper tree sections with smaller diameters may have limited the amount of food resources available and increased the potential for competition between larvae in a poorer quality environment for larval development, limiting their survival. In previous studies using cut logs, the highest mortality occurred in smaller logs having the highest density of neonates [5]. As trees used in this trial had lost their entire canopy following the drought collapse [36], leaving the upper sections of trees with thinner (<10 mm) bark to dry out quickly. This then meant there was insufficient time for borers to fully develop before sapwood became too dry, reducing their survival. This drying effect may also have reduced nutrient content as it is known to affect the performance of phytophagous insects [61]. It appears rapid drying influenced the decline in borer emergence of *P. semipunctata* with height, where a previous study showed low survival of *P. semipunctata* occurred when larvae began to develop in dry wood [62]. On the other hand, when *P. semipunctata* larvae are at high densities in other eucalypts, they develop quickly to avoid the problem of reduced nutrition in drier hosts where sapwood resources quickly became depleted [2,8].

## 5. Conclusions

Larger diameters and thicker bark in basal tree sections provided a better environment for larval development resulting in higher survival and greater borer emergence of *P. semipunctata*. Final densities of borers emerging from trees were largely controlled by the survival of neonate larvae where less than half did not reach maturity. This was attributed to upper tree sections having low survival and reduced incidence of larval feeding activity within the phloem/sapwood. This was a unique study that provided some insight into the natural behaviour of *P. semipunctata* attacking *Eucalyptus* trees in its native Australian habitat, where it is not normally considered a pest. The influence of bark thickness in defining the zone of larval activity where borer development is maximized may be useful for managing the invasiveness of *P. semipunctata* during attacks on *Eucalyptus* trees in exotic forests where the borer has become established.

## Figures and Tables

**Table 1:** Fitted models of diameter (cm) and bark thickness (mm) as a function of height (m), and bark thickness as a function of diameter for jarrah (*Eucalyptus marginata*) and marri (*Corymbia calophylla*) trees infested by the Australian eucalyptus longhorned borer *Phoracantha semipunctata* in the Northern Jarrah Forest (NJF), Southwestern Australia. Associations were analysed by least squares regression.

Associations	Species	Regression Equation	*p*-Value
Diameter × Height	Jarrah	*y* = −0.973*x* + 21.63	<0.001
	Marri	*y* = −1.372*x* + 22.76	<0.001
Bark thickness × Height	Jarrah	*y* = −0.720*x* + 12.56	<0.001
	Marri	*y* = −1.016*x* + 17.69	<0.001
Bark thickness × Diameter	Jarrah	*y* = −0.153*x* + 6.14	<0.001
	Marri	*y* = −0.595*x* + 3.69	<0.001

**Table 2:** Fitted models for borer emergence (m^−2^), incidence of larval galleries (%) and larval survival (%) of the Australian eucalyptus longhorned borer *Phoracantha semipunctata* as a function of height (m), diameter (cm) and bark thickness (mm) of jarrah (*Eucalyptus marginata*) and marri (*Corymbia calophylla*) trees in the Northern Jarrah Forest (NJF), Southwestern Australia. Associations were analysed by least squares regression.

Associations	Species	Regression Equation	*p*-Value
Borer emergence × Height	Jarrah	*y* = −0.700*x* + 16.36	<0.001
	Marri	*y* = −1.542*x* + 25.13	<0.001
Incidence × Height	Jarrah	*y* = −2.403*x* + 62.86	<0.001
	Marri	*y* = −4.561*x* + 75.81	<0.001
Larval survival × Height	Jarrah	*y* = −1.297*x* + 58.04	0.072
	Marri	*y* = −4.532*x* + 78.05	<0.001
Borer emergence × Bark thickness	Jarrah	*y* = 0.359*x* + 6.56	0.268
	Marri	*y* = 0.706*x* + 6.88	<0.001
Incidence × Bark thickness	Jarrah	*y* = 2.606*x* + 22.41	0.038
	Marri	*y* = 3.825*x* + 13.70	<0.001

**Table 3:** Tree factors influencing the distribution of borer emergence, borer emergence (m^−2^) and incidence of larval galleries (%) of the Australian eucalyptus longhorned borer *Phoracantha semipunctata* for jarrah (*Eucalyptus marginata*) and marri (*Corymbia calophylla*) trees in the Northern Jarrah Forest (NJF), Southwestern Australia. Predictions of the association (using Generalised Linear Mixed Models) between (**a**) number of borers emerged with height; (**b**) density of borers emerged with height and (**c**) incidence with height. The final analyses included all the variables and interactions with species and tree factors listed below (residual deviance; model 1 = 5.46, model 2 = 6.25, *n* = 861 degrees of freedom, residual deviance model 3 = 2.59, *n* = 454 degrees of freedom).

	Correlation Coefficient	SE	*F*-Value	*p*-Value
*(a) Number of borers emerged*				
Species	0.082	0.076	102.13	<0.001
Height	−0.298	0.106	59.28	<0.001
Diameter	0.506	0.008	949.40	<0.001
Species × Height	0.512	0.217	4.50	<0.001
Species × Diameter	0.140	0.010	1.48	0.224
Height × Diameter	−0.494	0.013	20.53	<0.001
Species × Height × Diameter	0.443	0.001	7.03	<0.001
*(b) Borer emergence (m^−2^)*				
Species	0.142	0.192	18.92	<0.001
Height	−0.266	0.458	3.8	<0.001
Diameter	0.264	0.018	21.22	<0.001
Species × Height	0.134	0.979	0.76	0.697
Species × Diameter	0.002	0.023	1.19	0.276
Height × Diameter	−0.476	0.045	2.21	0.01
Species × Height × Diameter	0.047	0.103	0.64	0.813
*(c) Incidence (%)*				
Species	0.024	0.017	3.36	0.067
Height	−0.277	0.297	3.92	<0.001
Diameter	0.365	0.015	129.62	<0.001
Species × Height	−0.016	9.408	0.44	0.853
Height × Diameter	−0.526	0.135	9.16	<0.001
Species × Diameter	−0.069	0.022	0.49	0.485
Species × Height × Diameter	0.045	1.600	1.46	0.187
